# Diagnostic accuracy of the Fibrosis-4 (FIB-4) index for advanced liver fibrosis in Qatari patients with MASLD: Comparison with liver biopsy and FibroScan

**DOI:** 10.5339/qmj.2026.32

**Published:** 2026-06-02

**Authors:** Moataz Derbala, Ibrahim M. Obeidat, Raya Abualsuod, Mahmoud Draidi, Nagah Selim, Jihene Maatoug, Alaa Elkalamawi

**Affiliations:** 1 Gastroenterology and Hepatology, Hamad Medical Corporation, Doha, Qatar; 2 Theodore Bilharz Research Institute, Warraq Al Hadar, Egypt; 3 Weill Cornell Medical College, Ar-Rayyan, Qatar; 4 Internal Medicine Department, Hamad Medical Corporation, Doha, Qatar; 5 Community and Preventive Medicine Department, Primary Health Care Corporation, Doha, Qatar; 6 Faculty of Medicine of Sousse, Sousse University, Sousse, Tunisia; 7 Epidemiology- Medicine, Lincoln University, Kota Bharu, Malaysia

**Keywords:** Fibrosis-4 index, FIB-4, MASLD, FibroScan, liver biopsy, accuracy

## Abstract

**Background:**

Metabolically-dysfunction-associated steatotic liver disease (MASLD) is highly prevalent in populations with metabolic risk factors, and the liver fibrosis stage is the main determinant of long-term outcomes. The Fibrosis-4 (FIB-4) index is widely recommended as a first-line noninvasive test for fibrosis risk stratification; however, its diagnostic performance may vary across populations.

**Objective:**

To evaluate the diagnostic performance of the FIB-4 index for detecting advanced liver fibrosis in Qatari residents with MASLD, using liver biopsy as the primary reference standard and FibroScan as a secondary comparator.

**Methodology:**

This retrospective, cross-sectional diagnostic accuracy study included adults with MASLD evaluated at Hamad Medical Corporation in Qatar between January 2023 and December 2024. FIB-4 was calculated from routine laboratory parameters, and fibrosis was staged using the METAVIR (Meta-Analysis of Histological Data in Viral Hepatitis) scoring system. It is a histological scoring system used to grade necroinflammatory activity (A0–A3) and stage liver fibrosis (F0–F4). Advanced fibrosis was defined as METAVIR stage ≥F3. Diagnostic performance metrics and the area under the receiver operating characteristic curve (AUROC) were calculated at established cut-offs.

**Results:**

A total of 117 patients were included; 74 patients (63.2%) underwent liver biopsy, and 91 patients (77.7%) underwent FibroScan assessment. Advanced fibrosis was present in 21 patients (17.9%) on biopsy. At a FIB-4 cut-off of 1.3, sensitivity and specificity for detecting advanced fibrosis were 66.7% and 67.9%, respectively, compared with liver biopsy, with an overall accuracy of 67.6% and a negative predictive value of 83.7%. The AUROC for FIB-4 was 0.76.

**Conclusion:**

FIB-4 can be used as a first-line, accountable screening tool, followed by another confirmatory tool in high-risk patients.

## 1. INTRODUCTION

Metabolically-dysfunction-associated steatotic liver disease (MASLD), previously referred to as nonalcoholic fatty liver disease (NAFLD), represents a rapidly growing global health burden and is now the most common chronic liver disease worldwide.^1^ The condition is linked to obesity, type 2 diabetes mellitus (T2DM), dyslipidemia, and other components of metabolic syndrome.^2^

The Middle East, including Qatar, carries a significant burden of metabolic disease. Population-based studies from primary care settings in Qatar have reported 48.8% prevalence of fatty liver disease, particularly among individuals with multiple metabolic risk factors.^3,4^ Given the rising prevalence of MASLD and its complications, early identification of patients at risk of advanced fibrosis has become a clinical and public health priority.

Liver biopsy remains the reference standard for staging liver fibrosis; however, its invasive nature, cost, and limited feasibility preclude its use as a large-scale screening tool.^5^ Consequently, noninvasive tests (NITs) have become a preference. Among these, the Fibrosis-4 (FIB-4) index, derived from age, aminotransferase levels, and platelet count, is widely recommended as a first-line screening tool due to its simplicity, low cost, and broad availability.^6^ Archer et al. endorse FIB-4 for initial fibrosis risk assessment in the European Association for the Study of the Liver (EASL).^7^

Despite its widespread use, the diagnostic performance of FIB-4 is known to vary across populations and clinical contexts. have shown that metabolic comorbidities, including obesity and diabetes, may affect FIB-4 accuracy.^8^ Ethnic, demographic, and regional differences may further affect liver enzyme distributions and fibrosis progression, underscoring the importance of population-specific validation.^9^

To date, the diagnostic accuracy of the FIB-4 index has not been systematically evaluated in the Qatari population according to our search in the Cochrane database and PubMed.^10^ Given the unique demographic characteristics, high prevalence of metabolic risk factors, and evolving MASLD care pathways in Qatar, local validation of FIB-4 against established reference standards is essential.^3,10^ Moreover, the increasing availability of disease-modifying therapies for MASLD has amplified the need for reliable, accessible tools that can effectively guide referrals and optimize healthcare resource utilization.^11^

The primary objective of this study was to evaluate the diagnostic performance of the FIB-4 index for detecting advanced liver fibrosis in Qatari residents with MASLD, using liver biopsy as the primary reference standard and vibration-controlled transient elastography (FibroScan) as a secondary comparator. Secondary objectives included assessing the concordance between FIB-4 and FibroScan and exploring the influence of metabolic risk factors, such as diabetes and obesity, on FIB-4 performance in this population.

## 2. METHODOLOGY

### 2.1 Ethical considerations

As this was a retrospective chart review, the requirement for informed consent was waived. All data were collected from electronic medical records in an anonymized form before analysis, and handled with strict confidentiality. Patient identifiers were removed at all stages of data extraction and analysis, and access to the dataset was restricted to the study investigators only. The study protocol MRC-01-25-1072 was reviewed and approved in November 2025 by the Institutional Review Board (IRB) at Hamad Medical Corporation (HMC), Doha, Qatar, in accordance with the Declaration of Helsinki and local ethical regulations.

### 2.2 Study design and population

This was a retrospective, cross-sectional diagnostic accuracy study conducted at the Hepatology Clinics of HMC, Qatar. The study included adult patients (≥18 years) with MASLD who underwent liver fibrosis assessment using liver biopsy and/or vibration-controlled transient elastography (FibroScan) between January 1, 2023, and December 31, 2024. A total of 117 patients were included. Liver biopsy was considered the primary reference standard for fibrosis staging, where available, while FibroScan was used as a secondary reference standard in patients who did not undergo biopsy.

The FIB-4 index was calculated for all included patients using laboratory parameters obtained closest in time to the reference fibrosis assessment. Consecutive patients were included using a retrospective sampling approach. An Excel sheet and an Access worksheet were used for data collection.

The FIB-4 score was calculated using the standard validated formula, developed by Sterling et al. on FIB-4 = (age [years] × AST [U/L])/(platelet count [10⁹/L] × √ALT [U/L]), where AST represents aspartate aminotransferase, ALT represents alanine aminotransferase, and platelet count is expressed as ×10⁹/L. 17

FIB-4 values were interpreted according to the established EASL guideline-recommended cut-offs. Values <1.3 were considered low risk for advanced fibrosis, values between 1.3 and 2.67 were considered intermediate risk, and values >2.67 were considered high risk for advanced fibrosis.^7^

### 2.3 Inclusion criteria

The study included adults aged ≥18 years with a diagnosis of fatty liver disease based on imaging and/or biopsy, who had available laboratory data required for calculation of the FIB-4 index and who underwent liver biopsy and/or FibroScan during the study period.

### 2.4 Exclusion criteria

Patients were excluded if they had viral hepatitis (HBV or HCV), autoimmune liver disease, significant alcohol consumption, a history of liver transplantation, established cirrhosis, or nonhepatic causes of thrombocytopenia.

### 2.5 Liver histological assessment

Liver biopsy specimens with a minimum length of 15 mm and containing at least six portal tracts were considered adequate for histological evaluation. Fibrosis staging was performed using the METAVIR (Meta-Analysis of Histological Data in Viral Hepatitis) scoring system, with stages defined as follows: F0, no fibrosis; F1, portal fibrosis without septa; F2, portal fibrosis with few septa; F3, bridging fibrosis with architectural distortion; and F4, cirrhosis. For diagnostic accuracy analyses, fibrosis stages were dichotomized into nonadvanced fibrosis (F0–F2) and advanced fibrosis (F3–F4).

### 2.6 FibroScan assessment

FibroScan was performed according to standard clinical protocols. According to Berger et al. liver stiffness measurement (LSM) values were interpreted using established thresholds, with values <7.0 kPa indicating no or mild fibrosis, 7.0 to 9.5 kPa indicating significant fibrosis, 9.5 to 12.5 kPa indicating advanced fibrosis, and values >12.5 kPa indicating cirrhosis.^12^

### 2.7 Reference standards and outcomes

The primary outcome was the diagnostic accuracy of FIB-4 for detecting advanced liver fibrosis (≥F3) using liver biopsy as the reference standard.

Secondary analyses evaluated the performance of FIB-4 against FibroScan-derived LSMs in patients without available biopsy data.

### 2.8 Statistical analysis

Descriptive statistics were used to summarize baseline characteristics of the study population. Continuous variables were expressed as mean ± standard deviation when normally distributed, and as median with interquartile range when nonnormally distributed. Categorical variables were presented as frequencies and percentages.

Comparisons between categorical variables were performed using the chi-square test, as appropriate. A two-sided P-value < 0.05 was considered statistically significant.

The diagnostic performance of the FIB-4 index was evaluated by calculating sensitivity, specificity, positive predictive value (PPV), negative predictive value (NPV), and overall accuracy using liver biopsy and FibroScan as reference standards. Receiver operating characteristic (ROC) curve analysis was performed to assess the discriminative ability of FIB-4 and to identify optimal cut-off values for predicting advanced liver fibrosis in the Qatari population. The area under the ROC curve (AUROC) was used as a summary measure of diagnostic accuracy.

All statistical analyses were performed using PSPP Statistics software.

## 3. RESULTS

Baseline demographic and clinical characteristics are summarized in Table 1. Among the study cohort, 49 patients (41.9%) had diabetes mellitus, 51 patients (43.6%) were obese, and 29 patients (24.8%) had both diabetes and obesity. Note that the majority of patients had dyslipidemia (67, 57.3%) and hypertension (35, 29.9%; Table 1).

Based on liver biopsy findings, fibrosis stages were distributed as follows: F0 in 21 patients (17.9%), F1 in 32 patients (27.4%), F2 in 8 patients (6.8%), F3 in 3 patients (2.6%), and F4 in 10 patients (8.5%). Overall, 13 patients (11.1%) had advanced fibrosis (≥F3) on biopsy. According to FibroScan, fibrosis stages were categorized as none to mild fibrosis in 76 patients (76.0%) and moderate to severe fibrosis in 15 patients (12.8%). Using the FIB-4 index, 75 patients (64.1%) were classified as low risk, 32 patients (27.4%) as intermediate risk, and 10 patients (8.5%) as high risk for advanced fibrosis (Table 1).

The diagnostic performance of the FIB-4 index for detecting advanced liver fibrosis was evaluated using liver biopsy as the primary reference standard and FibroScan as a secondary reference standard. At the 1.3 cut-off, FIB-4 demonstrated a sensitivity of 66.67% and specificity of 67.92% for detecting advanced fibrosis compared with liver biopsy. When FibroScan was used as the reference standard, the sensitivity and specificity at the same cut-off were 60.0% and 78.43%, respectively. The overall diagnostic accuracy of FIB-4 was 67.57% when validated against liver biopsy and 70.33% when validated against FibroScan (Table 2). ROC curve analysis showed an area under the curve (AUROC) of 0.76, indicating acceptable discriminative ability of FIB-4 for identifying advanced fibrosis.

Cross-tabulation analyses demonstrated moderate concordance between FIB-4 and results (Table 3). Among patients classified as having non-advanced fibrosis by FIB-4, 36 (48.6%) had non-advanced fibrosis on biopsy, whereas 7 (9.5%) had advanced fibrosis. Among patients classified as having advanced fibrosis by FIB-4, 14 (18.9%) were confirmed to have advanced fibrosis on biopsy. Similarly, comparison between FIB-4 and FibroScan showed concordant classification in a substantial proportion of patients, with 40 patients (44.0%) classified as non-advanced fibrosis and 24 patients (26.4%) classified as advanced fibrosis by both modalities (Tables 4–12).

Discrepancies between FIB-4 and liver biopsy results were further explored across metabolic subgroups. Among patients with diabetes mellitus, 61 (52.4%) were classified as having fibrosis using FIB-4, compared with 35 (30.2%) identified by liver biopsy. Among obese patients, fibrosis was identified in 56 (47.9%) using FIB-4 versus 37 (32.0%) by liver biopsy. In patients with both diabetes and obesity, fibrosis was identified in 33 (28.6%) using FIB-4, compared with 17.7 (15.1%) using liver biopsy.

## 4. DISCUSSION

Metabolically-dysfunction-associated steatotic liver disease (MASLD) is increasingly recognized as a major public health concern, largely driven by the global rise in obesity, diabetes mellitus, and other metabolic risk factors.^8,13^ Liver fibrosis stage remains the most important determinant of prognosis in MASLD, with advanced fibrosis being associated with an increased risk of liver-related complications, hepatocellular carcinoma, and mortality.^1^ Early identification of patients with advanced fibrosis is critical to guide appropriate referral, surveillance, and therapeutic decision-making.^11^

In this study, we evaluated the diagnostic performance of the FIB-4 index for the detection of advanced liver fibrosis in a cohort of Qatari residents with MASLD, using liver biopsy as the primary reference standard and FibroScan as a secondary noninvasive comparator. Our findings demonstrate that FIB-4 shows acceptable diagnostic performance, with sensitivity and NPV supporting its role as a screening and rule-out tool for advanced fibrosis rather than as a definitive diagnostic test. The principal clinical value of FIB-4 in this cohort lies in its ability to exclude advanced fibrosis, while positive or indeterminate results require confirmatory second-line testing.

When validated against liver biopsy, FIB-4 achieved a sensitivity of 66.7% and an NPV of 83.7% at the conventional cut-off of 1.3, indicating that a low FIB-4 score reliably excludes advanced fibrosis in a substantial proportion of patients.^13–15^ Similar, albeit slightly lower, performance was observed when FibroScan was used as the reference standard. These findings are consistent with the published meta-analyses by Han et al. and large cohort studies done by Lin et al. which have reported AUROC values for FIB-4 ranging from approximately 0.70 to 0.80 for the detection of advanced fibrosis in MASLD and NAFLD populations.^8^

Importantly, our results align with current international clinical practice guidelines, including those from EASL, which recommend FIB-4 as a first-line NIT for fibrosis risk stratification. Agreeing that patients with a FIB-4 score below 1.3 are considered at low risk for advanced fibrosis and can be safely managed in primary care with periodic reassessment, whereas patients with a FIB-4 score ≥1.3 should undergo second-line testing, such as vibration-controlled transient elastography, to refine fibrosis risk assessment.^7^ Our findings support the applicability of this stepwise approach within the Qatari healthcare setting.^3^

The moderate concordance observed between FIB-4 and liver biopsy, as well as between FIB-4 and FibroScan, reflects known limitations of serum-based biomarkers, particularly in metabolically complex populations.^1,8^ In subgroup analyses, FIB-4 tended to classify a higher proportion of patients with diabetes, obesity, dyslipidemia, and hypertension as having fibrosis compared with histological assessment. This observation is consistent with existing recommendations suggesting that FIB-4 may overestimate fibrosis risk in patients with metabolic comorbidities, likely due to age, aminotransferase levels, and platelet counts in the score.^11^

To our knowledge, this is the first study to assess the performance of the FIB-4 index in a Qatari MASLD population. Given the high prevalence of metabolic risk factors in Qatar and the wider Middle East, these findings highlight the need for population-specific validation of noninvasive fibrosis assessment tools. Our findings suggest that FIB-4 retains its utility as an initial triage tool in this population, with diagnostic performance comparable to that reported in international cohorts. Additionally, confidence intervals for diagnostic accuracy measures were not calculated due to the relatively small sample size, and subgroup analyses were exploratory in nature.

Clinically, implementing a FIB-4-based triage strategy in Qatar helps optimize referral pathways by identifying low-risk patients suitable for follow-up in primary care, while prioritizing higher-risk individuals for elastography and specialist evaluation. This approach reduces unnecessary referrals and improves allocation of hepatology resources in a high-burden metabolic population.

## 5. CONCLUSION

FIB-4 is a simple, inexpensive, and noninvasive tool with acceptable performance for excluding advanced fibrosis in Qatari patients with MASLD. Its use as a first-line screening test, followed by second-line elastography in patients with indeterminate or high-risk scores, optimizes referral pathways, reduces invasive procedures, and improves risk stratification in routine clinical practice.

## ACKNOWLEDGMENTS

The author(s) have no acknowledgements to declare.

## FUNDING

The author(s) received no financial support for the research, authorship, and/or publication of this article.

## CONFLICT OF INTEREST

The author(s) declare that there is no conflict of interest.

## AUTHOR CONTRIBUTIONS

To determine authorship, we followed the guidelines of the International Committee of Medical Journal Editors (ICMJE). Each author provided sufficient contribution to the concept of the work: MD, IMO, RA, MD, NS, JM, and AE: data acquisition; MD, IMO, JM, and AE: analysis; MD, IMO, and AE: interpretation; IMO, MD, and AE: worked on drafting and critically revising the manuscript for important intellectual content. All authors approved the version to be published.

## DATA AVAILABILITY STATEMENT

Data can be obtained from the corresponding author upon request. Ethical approval MRC-01-25-1072 was reviewed and approved in November 2025.

## DISCLOSURE OF AI USE

The authors declare that no artificial intelligence (AI) tools were used in the preparation of this manuscript.

## Figures and Tables

**Table 1.** Demographics of population.

Characteristic variable	Count (*n* = 117)	Percentage (%)
**Nationality**		
Qatari	32	27.4
Others	85	72.6
**Obesity**		
No	63	53.8
Yes	51	43.6
**Diabetes mellitus diagnosis**		
No	68	58.1
Yes	49	41.9
**Obesity and DM**		
No	88	75.2
Yes	29	24.8
**Dyslipidemia**		
No	50	42.7
Yes	67	57.3
**Hypertension**		
No	82	70.1
Yes	35	29.9
**Fibrosis stages by liver biopsy**		
F0	21	17.9
F1	32	27.4
F2	8	6.8
F3	3	2.6
F4	10	8.5
**Fibrosis stages by FibroScan**		
F1	51	43.6
F2	25	32.4
F3	9	7.7
F4	6	5.1
**FIB-4 category**		
F1 (mild)	75	64.1
F2 (moderate)	32	27.4
F3 (severe)	10	8.5

**Table 2.** Performance of FIB-4 in detecting fibrosis compared to liver biopsy and FibroScan, respectively.

Reference standard	FIB-4 cut-off	Sensitivity (%)	Specificity (%)	PPV (%)	NPV (%)	Accuracy (%)
Liver biopsy	≥1.3	66.67	67.92	45.16	83.72	67.57
FibroScan	≥1.3	60.00	78.43	68.57	71.43	70.33

Performance metrics were calculated for the detection of advanced fibrosis (F3–F4). The cut-off of 1.3 was used as a rule-out threshold in accordance with guideline recommendations.

**Table 3.** Comparison of FIB-4 categories with liver biopsy fibrosis stage.

FIB-4 classification (index test)	Nonadvanced fibrosis (F0–F2) liver biopsy	Advanced fibrosis (F3–F4) liver biopsy	Total
Nonadvanced fibrosis	36 (48.6%)	7 (9.5%)	43 (58.1%)
Advanced fibrosis	17 (23.0%)	14 (18.9%)	31 (41.9%)
Total	53 (71.6%)	21 (28.4%)	74 (100%)

**Table 4.** Correlation between FIB-4 and FibroScan results.

FibroScan fibrosis stage	FIB-4: nonadvanced fibrosis	FIB-4: advanced fibrosis	Total
Nonadvanced fibrosis	40 (44.0%)	11 (12.1%)	51 (56.0%)
Advanced fibrosis	16 (17.6%)	24 (26.4%)	40 (44.0%)
Total	56 (61.6%)	35 (38.4%)	91 (100%)

**Table 5.** Diabetic and obese.

	Frequency	Percent	Valid percent	Cumulative percent
**Valid**				
0	88	75.2%	75.2%	75.2%
1	29	24.8%	24.8%	100.0%
Total	117	100.0%		

0 is no diabetes or obesity,1 diabetes and obesity.

**Table 6.** Summary.

	Cases					
	Valid		Missing		Total	
	*N*	Percent	*N*	Percent	*N*	Percent
Obese (BMI = 30) × Diabetes mellitus	115	98.3%	2	1.7%	117	100.0%

**Table 7.** Obese (BMI = 30) × diabetes mellitus (yes or no).

	Diabetes mellitus (yes or no)		Total
	0	1	
**Obese (BMI = 30)**			
** 0**			
Count	43	20	63
Row %	68.3%	31.7%	100.0%
Column %	65.2%	40.8%	54.8%
Total %	37.4%	17.4%	54.8%
** 1**			
Count	23	28	51
Row %	45.1%	54.9%	100.0%
Column %	34.8%	57.1%	44.3%
Total %	20.0%	24.3%	44.3%
** 25**			
Count	0	1	1
Row %	0%	100.0%	100.0%
Column %	0%	2.0%	0.9%
Total %	0%	0.9%	0.9%
** Total**			
Count	66	49	115
Row %	57.4%	42.6%	100.0%
Column %	100.0%	100.0%	100.0%
Total %	57.4%	42.6%	100.0%

0 is no existing condition,1 existing condition, 5 existing over weight.

**Table 8.** Chi-square test.

	Value	df	Asymptotic significance (2-tailed)
Pearson chi-square	7.54	2	0.023
Likelihood ratio	7.95	2	0.019
Linear-by-linear association	2.77	1	0.096
Number of valid cases	115		

**Table 9.** Liver biopsy.

	Frequency	Percent	Valid percent	Cumulative percent
**Valid**				
0.00	53	45.3%	71.6%	71.6%
1.00	21	17.9%	28.4%	100.0%
Missing	43	36.8%		
Total	117	100.0%		

**Table 10.** Case summary.

Liver biopsy	Valid *N* (listwise)	
	Unweighted	Weighted
Positive	21	21.00
Negative	53	53.00

**Table 11.** Area under the curve.

Variable under test	Area
FIB_4_Score_Auto_c	0.76

**Table 12.** Coordinates of the curve.

Test variable	Positive if greater than or equal to	Sensitivity	1 − specificity
FIB_4_Score_Auto_c	−0.78169681	1.00	1.00
	0.51849522	1.00	0.87
	0.56800940	1.00	0.83
	0.62090276	1.00	0.81
	0.65100676	0.95	0.79
	0.70000000	0.95	0.77
	0.73093042	0.90	0.72
	0.75359813	0.90	0.70
	0.76473700	0.90	0.68
	0.80621132	0.90	0.62
	0.81094667	0.90	0.62
	0.82037354	0.90	0.60
	0.83472277	0.90	0.58
	0.83766487	0.90	0.58
	0.84742788	0.90	0.58
	0.85349002	0.90	0.58
	0.85355595	0.90	0.58
	0.86696039	0.90	0.57
	0.86947409	0.90	0.57
	0.88851695	0.90	0.57
	0.90378011	0.86	0.51
	0.90437768	0.86	0.51
	0.93465569	0.86	0.45
	0.97296975	0.86	0.43
	1.01122745	0.86	0.40
	1.03253892	0.86	0.40
	1.03300555	0.86	0.40
	1.04119462	0.86	0.38
	1.06828200	0.86	0.38
	1.07718583	0.86	0.38
	1.07740087	0.86	0.38
	1.10799990	0.86	0.36
	1.11858810	0.86	0.36
	1.18616775	0.76	0.36
	1.20000000	0.76	0.36
	1.20809803	0.76	0.36
	1.22719491	0.76	0.36
	1.23031493	0.76	0.36
	1.23557785	0.76	0.34
	1.23580723	0.76	0.34
	1.30290418	0.71	0.32
	1.43561007	0.57	0.28
	1.44990145	0.57	0.28
	1.47474443	0.57	0.26
	1.48500000	0.57	0.26
	1.51458203	0.57	0.25
	1.55123307	0.57	0.25
	1.55897200	0.57	0.23
	1.56606005	0.57	0.23
	1.57731404	0.57	0.23
	1.58024106	0.57	0.23
	1.60000000	0.57	0.23
	1.66096882	0.52	0.23
	1.70395748	0.52	0.23
	1.75000000	0.52	0.23
	1.83365735	0.52	0.23
	1.91831747	0.52	0.23
	2.04772751	0.48	0.19
	2.20777341	0.43	0.13
	2.23027977	0.43	0.11
	2.25802560	0.43	0.11
	2.35239667	0.38	0.09
	2.47318744	0.33	0.08
	2.51016761	0.29	0.08
	2.90676720	0.24	0.06
	2.95427363	0.24	0.04
	3.07280746	0.19	0.04
	3.35000000	0.19	0.04
	3.55000000	0.19	0.04
	3.92675584	0.14	0.04
	5.32526023	0.10	0.02
	5.38977700	0.10	0.02
	10.00474557	0.00	0.02
	10.07055129	0.00	0.02
	20.22664817	0.00	0.00
